# Diabetes leading to heart failure and heart failure leading to diabetes: epidemiological and clinical evidence

**DOI:** 10.1007/s10741-022-10238-6

**Published:** 2022-05-06

**Authors:** Alberto Palazzuoli, Massimo Iacoviello

**Affiliations:** 1 Cardiovascular Diseases Unit, Cardio-Thoracic and Vascular Department, S. Maria Alle Scotte Hospital, University of Siena, Siena, Italy; 2 Department of Medical and Surgical Sciences, University of Foggia, Via Luigi Pinto 1, 71121 Foggia, Italy

**Keywords:** Type 2 diabetes mellitus, Heart failure, Epidemiology, Prognosis

## Abstract

Type 2 diabetes mellitus (T2DM) is a risk factor that plays a major role in the onset of heart failure (HF) both directly, by impairing cardiac function, and indirectly, through associated diseases such as hypertension, coronary disease, renal dysfunction, obesity, and other metabolic disorders. In a population of HF patients, the presence of T2DM ranged from 20 to 40%, according to the population studied, risk factor characteristics, geographic area, and age, and it is associated with a worse prognosis. Finally, patients with HF, when compared with those without HF, show an increased risk for the onset of T2DM due to several mechanisms that predispose the HF patient to insulin resistance. Despite the epidemiological data confirmed the relationship between T2DM and HF, the exact prevalence of HF in T2DM comes from interventional trials rather than from observational registries aimed to prospectively evaluate the risk of HF occurrence in T2DM population. This review is focused on the vicious cycle linking HF and T2DM, from epidemiological data to prognostic implications.

## Introduction

Diabetes mellitus is a well-established risk factor for a wide range of cardiovascular (CV) events, including macrovascular and microvascular complications [1, 2]. The prevalence and incidence of diabetes mellitus, and type 2 DM (T2DM) in particular, in patients with heart failure (HF), and the related risks associated with this condition, have not been fully described in a contemporary population [3].

The recent advances in diabetic treatment further highlight the need for better understanding of the complex relationship between T2DM and HF. Subjects affected by T2DM have a higher incidence of HF and a worse outcome than non-diabetic people [4]. T2DM is associated with the onset of HF both directly and indirectly through associated CV risk factors and diseases.

In many studies, HF is considered a CV complication. However, up to now, few studies have directly analyzed the real impact of T2DM in HF, and most reports describe post hoc or retrospective data [5–7]. Altered glycemic status is often associated with other CV risk factors and metabolic disorders. Therefore, T2DM may accelerate coronary and systemic atherosclerosis, endothelial damage, autonomic dysfunction, and extracellular matrix collagen deposition [8]. In addition to these factors, diabetic cardiomyopathy [9, 10] represents another potential cause of HF development, independent of the presence of vascular disease. Additionally, HF may increase the incidence of T2DM, whose onset is associated with a worse outcome [11].

The aim of this review is to describe epidemiological aspects and potential causal factors that explain the dangerous relationship between T2DM and HF.

## Diabetes in heart failure

### Diabetes as potential HF risk factor

**Fig. 1:**
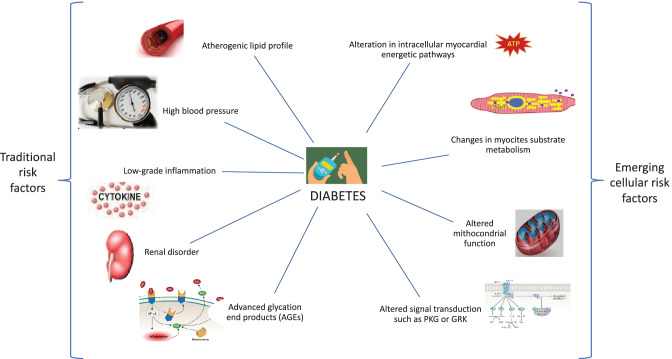
Traditional and emerging risk factors that explain the increased risk of adverse events in patients with HF and associated diabetes

Altered glycemic status is often associated with other CV risk factors, such as hypertension, obesity, and dyslipidemia. These factors have been highlighted in the new definition of HF, and they represent initial risk factors for HF occurrence [12]. Therefore, T2DM per se, may accelerate coronary and systemic atherosclerosis, vascular alteration, autonomic dysfunction, and extracellular matrix collagen deposition [8]. This relationship may confound the risk assessment, and it is unclear how much the adverse outcome is directly due to glycemic dysfunction or linked to the higher risk burden. T2DM is also associated with an atherogenic lipid profile, high blood pressure, low-grade inflammation, renal disorder, and advanced glycation end products (AGEs), all of which increase CV risk [13, 14]. The altered endothelial function is associated with an adverse lipid profile and vascular inflammation, which produce pro-atherogenic signals (P selectin, vascular grow factors, transforming grow factor β) that are responsible for systemic vascular damage. Both arterial and cellular alterations lead to increased myocardial susceptibility, fibrosis, and collagen deposition, mediated by energetic substrate modification and altered intracellular signals. Among all patients affected by T2DM, about 90% are overweight, 70% are dyslipidemic, and almost 70% suffer from hypertension [7] (Fig. 1).

In the trial EMPAREG OUTCOME, 65% of patients had a prior myocardial infarction or stroke, and 12%, 40%, 30%, and 18% were at low, intermediate, high, and very high cardiovascular risk according to TIMI Risk Score for Secondary Prevention [15]. In the DECLARE Study, more than 90% of patients had multiple risk factors for cardiovascular disease or had established atherosclerotic CV disease defined as evidence of ischemic heart disease, ischemic cerebrovascular disease, or peripheral artery disease [16]. In the previously published ORIGIN trial, 59% of diabetic patients had cardiovascular event, 35% MI, and 79% affected by hypertension [17]. Moreover, more than half took statins and 80% antihypertensive or diuretic agents. Finally, in a post hoc analysis of the LEADER trial comparing patients with and without HF, the distribution of CV drugs was similar independently on NYHA class and the CV risk profile was comparable in all subgroups [18]. Current findings could explain several gaps in evaluating the risk burden and related risk for HF in this setting.

As far as functional status and exercise capacity are concerned, T2DM is characterized by a reduced exercise tolerance, impaired quality of life, and poor life expectancy due to both CV and peripheral muscle alterations independently on the presence of HF. In the cross-sectional cohort study, T2DM was much more common in women than in men, and its prevalence increased linearly as patient age from 46 to 74 years [2]. In this population, HF risk was twofold and fivefold greater in diabetic men and women, respectively, compared to non-diabetic men and women. In a meta-analysis of several cardiovascular outcome trials in T2DM patients, the authors found that HF was one of the most frequent nonfatal CV events, second only to myocardial infarction, with a mean prevalence ranging from 13 to 30% [19]. The wide range reported is due to the heterogeneity in HF diagnosis, the broad HF definition used, the lack of HF subtypes recognition, poor differentiation between chronic stable HF and de novo episodes, and population characteristics. In the ARIC study evaluating baseline diabetic patients without evidence of cardiac dysfunction, incidence of HF was strictly related with the presence of coronary artery disease (CAD) and it increases for each 1% higher glycosylated hemoglobin (HbA1c): 1.17 for the non-CAD group and 1.20 (CI 1.04–1.40) for the CAD group [20]. Similarly, in the Reykjavik study, across 20 years of mean follow-up, HF was diagnosed in 3.2% of non-diabetic patients compared with 6.0 and 11.8% among those with glycemic alteration and T2DM, respectively [21]. More recently, it has been demonstrated that not only the T2DM presence but also the changes in HbA1c over time were independently associated with risk of incident HF among patients with T2DM [22].

### Prevalence of T2DM in HF population and related risk

Over the last two decades, the prevalence of T2DM in HF patients has gradually increased from 12 to 22% in people 70 years or older [13, 23]. The current trend may be the result of the increased incidence of T2DM in the general population, improvement of diagnostic care, population aging, and prevention programs diffusion in industrialized countries [24]. An observational study reported that 12% of diabetic patients admitted to the hospital have HF, and those without HF had a 3.3% increased risk per year for disease developing [3].

In addition to these reports, few studies have directly analyzed the real impact of T2DM in HF, and most of the reports have described post hoc or retrospective data [5–7]. The more intriguing data come from the recent introduction of sodium glucose transport inhibitors (SGLT2-i), which have a positive effect on both CV- and HF-related events [25, 26].

In clinical trials, prevalence of T2DM in HF population varies according to the presence of chronic kidney disease (CKD), the coexistence of other CV diseases, and the duration of the glyco-metabolic disorder. In the observational Rotterdam study, 28% of diabetic patients had HFrEF, and 25% experienced HFpEF [27]. In the pilot ESC survey, 30% of HF diabetic patients had a preserved EF [28]. Moreover, a post hoc analysis from CHARM revealed that the prevalence of T2DM in HFrEF is 28.5% vs 28.3% in those with HFpEF [29]. T2DM prevalence also varies across race and lifestyle. In a cohort analysis from two different registries, HF-ACTION and ASIAN HF, the authors showed relevant differences with the lowest T2DM prevalence in Whites (29.3%), followed by Japanese/Koreans (34.1%), Blacks (35.9%), Chinese (42.3%), and Indians (44.2%); the highest prevalence is in Malays (51.9%) [30]. As previously reported, most of the patients with HF and preserved left ventricular ejection fraction (HFpEF) have glycaemic intolerance, increased body mass index, or other metabolic disorders [31, 32]. In addition to these factors, T2DM can be associated with diabetic cardiomyopathy by a direct damage on myocytes and endothelial cells due to an overexpression of end-glycosylated product, collagen deposition, and augmented inflammatory pathways. Diabetic cardiomyopathy type is distinguished for increased left ventricular (LV) mass and volume, impaired left ventricular LV filling, diastolic dysfunction, and reduced arterial compliance [9, 10]. Since T2DM is often associated with the above cited metabolic disorders, an emerging syndrome called metabolic cardiomyopathy is now acknowledged. This is the final expression of multiple metabolic and cellular disorders that lead to ventricular hypertrophy and stiffness, reduced arterial and ventricular compliance, and increased filling pressure and afterload [31–33].

T2DM can affect myocardial structure and function by several mechanisms, among which the most important is related to insulin resistance of muscle, liver, and pancreatic cells. The lack of insulin response in these systems leads to systemic glucotoxicity, fatty acid lipotoxicity, accelerated lipolysis, increased renal glucose absorption, and reduced incretin levels from gastrointestinal tract. Of note, the cardiovascular damage may arise from different alterations including metabolic (lipogenesis and gluconeogenesis), renal (increasing Na and glucose resorption), myocardial (sarcomeric stiffness and fibrosis overexpression), endothelial (increased RAA activity vascular grow factors, reduced NO synthase), and inflammatory disorders (increased expression of interleukines facilitating thrombogenesis). The prevalence of each pathophysiological mechanism may explain the different HF pattern and cardiac structural adaptation. Behind these metabolic and energetic dysfunctions, patients with glycaemic associated metabolic disorders, and their conditions cannot be controlled with common antidiabetic drugs [34, 35]. Coronary artery disease, left ventricular hypertrophy, and CKD are common associated diseases that explain the worse outcome observed in these patients. Current overlapping makes difficult the distinction between cardiac structural abnormalities related to primitive diabetic damage or associated conditions.

### HF prevalence and outcomes in the diabetic population

Several trials suggested T2DM begets HF and vice versa. The prevalence of HF in diabetic population is wide and it is related to HF definition and inclusion criteria that are often misleading [3, 4]. Of note, interventional trials demonstrated a mild reduction in CV events with glucose-lowering treatment if associated with other lifestyle modification and dietary programs, but the risk for HF remains increased [36, 37]. In a Scottish echocardiographic study, 71% of diabetic subjects experienced LV hypertrophy and 41% had diastolic dysfunction [38]. LV is associated with increased collagen expression and extracellular fibrotic expansion, leading to increasing cardiac stiffness, reduced longitudinal contraction, and increased vascular shear stress [39–41]. Notably, a retrospective analysis from diabetic patients with HFpEF showed increased LV thickness, left atrial enlargement, reduced quality of life associated with a higher rate hospitalization, and CV death, compared to non-diabetic subjects [42]. In this study, the authors observed a U-shaped trend in HbAc1, with the lowest risk of death in patients with moderate glucose control and higher risk in those with the optimal HbAc1 level; this suggests that simple reduction of glycemic levels is not the optimal treatment [43, 44].

A number of studies have also demonstrated the worse outcome of HF patients affected by T2DM. The increased all-cause mortality of HF patients affected by T2DM show some differences related to geographical area, population characteristics, and disease severity. In northern Europe and US registries, the prevalence of T2DM in HF patients is around 20%, whereas two studies conducted in different European countries reported a significant higher rate of T2DM in HF patients, despite lower body mass and younger age [28, 45–47]. Additionally, the prevalence of cardiac dysfunction and early stage of HF in asymptomatic diabetic patients ranges between 30 and 40%, and it is more prevalent in women and older subjects [48, 49]. Patients with T2DM have a 30% greater risk of requiring hospitalization for acute HF than do patients without T2DM. These patients experience a threefold greater risk of hospitalization for recurrent decompensation. The REACH study demonstrates an increased risk for hospitalization (HR 4.73) and a higher risk for CV mortality (HR 2.45) in patients with both HF and T2DM [50]. In the Swedish HF registry, absolute mortality is 48% in women with T2DM vs 40% in women without T2DM, and mortality rates in males are 43% and 35%, respectively [51]. Similarly, the Rotterdam study found that 17.5% of HF patients had T2DM and they revealed an increased risk for CV mortality during a mean 6.2 years follow-up (HR 3.19) [27]. In the post hoc analysis from CHARM, the adjusted HR for combined risk of mortality and hospitalization showed an increased HR both in HFrEF (1.64) and HFpEF (2.04) when compared to non-diabetic patients [29]. The more recent PARADIGM trial reveals that a consistent percentage of patients with T2DM (35%) have a higher risk of the primary composite outcome of HF hospitalization and cardiovascular mortality compared with those without a history of T2DM (HR 1.38) [52]. The long-term HFA registry [28] reveals higher cumulative rates of 1-year CV mortality (HR 1.28) and HF hospitalization (HR 1.37) independent of other risk factors. The mortality risk appears to be similarly independent of HF etiology and subtype. T2DM is associated with greater risk of CV mortality and HF hospitalization in subjects with HFpEF compared to those with HFrEF (HR 2.0 vs 1.6). The rates of adverse CV events per 1000 patients/years are 119 and 75 for diabetic and non-diabetic patients with low EF, respectively. For HFpEF, the rates are 58 and 31 for diabetic and non-diabetic patients, respectively [36]. In the I-PRESERVE study, hospitalization for HF occurred in 34% of diabetic patients vs 22% of those without diabetes, and diabetics had an increased HR mortality risk of 1.59 [53] (Table 1).

**Table 1:** Main large scale studies evaluating the prognostic impact of diabetes in patients with chronic heart failure

**Clinical trial**	**Year of publication**	**Type of study**	**Follow-up**	**Total patients** **(*n*)**	**Main findings**
**HFrEF and HFpEF trials**					
Incidence of Heart Failure in 2,737 Older Persons With and Without Diabetes Mellitus [48]	1999	Observational prospective	43 month	2727	Diabetes mellitus (*p* = 0.0003, risk ratio = 1.337) was significantly positively associated with the time to the development of CHF
The Rotterdam Study [27]	2001	Observational	6.1year	5255	Adjusted all-cause mortality risk of T2DM: HR = 3.19
High prevalence of previously unknown heart failure and left ventricular dysfunction in patients with type 2 diabetes [52]	2012	Cross-sectional study		605	161 (27.7%) have unknown heart failure: 28 (4.8%) with reduced ejection fraction, and 133 (22.9%) with preserved ejection fraction. The prevalence of heart failure increased steeply with age
Risk factors, treatment and prognosis in men and women with heart failure with and without diabetes [51]	2015	Observational	Median : 1.9 years	36,274	T2DM was a stronger predictor of mortality in women (OR 1.72) than in men (OR 1.47)
ESC-HFA HF Long-Term Registry [28]	2017	Prospective	1-year	9,428	Diabetic had higher cumulative rates all-cause death (9.4% vs. 7.2%; HR 1.28), CVD death (4.8% vs. 3.8%; adjusted HR 1.28), and HF hospitalization (13.8% vs. 9.3%HR 1.37)
CHARM programme [29]	2008	Interventional Candesartan	4-year	7599	Diabetes was associated with a greater relative risk of CV death or HF hospitalization in patients with preserved EF (HR 2.0) than in patients with low EF (HR 1.60)
GISSI-HF [46]	2017	Observational	3.9 years	6935	DM was associated with an increased risk of all-cause death (HR 1.43); higher HbA1c associated with increased risk of all-cause death: HR 1.21; and composite end point: HR 1.14
Prognostic Impact of Diabetes on Long-term Survival Outcomes in Patients With Heart Failure: A Meta-analysis [54]	2017	Combined randomized and observational studies	More than 1-year	381,725	Diabetes was associated with a higher risk of all-cause death (random-effects hazard ratio [HR 1.28], cardiovascular death [HR 1.34], and hospitalization [1.35])
**HFrEF trials**					
PARADIGM-HF [52]	2016	Interventional Sacubitril/valsartan	3 years	8399	The HR for patients with undiagnosed T2DM and known T2DM compared with those with HbA1c < 6.0% was 1.39; *P* < 0.001 and 1.64 (1.43–1.87); P < 0.001, respectively. Patients with pre-diabetes were also at higher risk (HR, 1.27)
**HFpEF trials**					
I-PRESERVE [53]	2017	Interventional Irbesartan	56 months	4128	Adjusted all-cause mortality risk of T2DM HR: 1.59

Finally, a meta-analysis including 12 trials with both acute and chronic HF patients and different EF thresholds confirms that T2DM is associated with increased risk of CV mortality (HR 1.34) and combined endpoint of mortality and HF hospitalization (HR 1.41) during a mean follow-up period of 3 years [54]. The increased risk of CV mortality is strictly related to baseline conditions and an increased CV risk burden. Most of these data come from HF registries, whereas studies in T2DM are often affected by several gaps making HF definition and occurrence inconsistent [55, 56]. The proportion of HF patients varied between 10 and 27% according with the criteria used to assess its presence. Most of studies reported NYHA class but did not HF treatment. Most of the trials included HF events among all the other MACE, without a pre-specified analysis. Finally, although the last studies considered the occurrence of HF hospitalization as event, they did not provide analyses about other end-points such as outpatients’ HF worsening or changes in their HF treatment [55, 56].

Additionally, patients with T2DM have worse NYHA class, reduced exercise tolerance, and peak oxygen uptake [57]. Finally, patients in the early stages of HF and LV dysfunction are more likely to progress to a symptomatic state compared to non-diabetics, and they experience higher congestion score and increased myocardial damage, as assessed by traditional laboratory screening (i.e., troponin and natriuretic peptides) [58, 59].

## Heart failure leading to diabetes

The pathophysiological background underlying the greater incidence of T2DM in HF patients is complex and mainly related to the presence of insulin resistance in HF patients [60, 61], even in the absence of pathologically elevated fasting glucose plasma levels [62]. Insulin resistance is determined by different factors, including neuro-hormonal activation. In particular, sympathetic nervous system overactivity plays a key role in the development of insulin resistance [63, 64]. This could be due to the stimulation of alpha-adrenergic receptors by catecholamines, a process that causes vasoconstriction and hypoperfusion at the level of skeletal muscle and is responsible for reduced glucose uptake [64]. Catecholamine-mediated insulin resistance could be also related to increased oxidative stress [63], lipolysis, and availability of free fatty acids availability, as well as to reduced skeletal muscle glucose uptake, glucose metabolism, and increased hepatic gluconeogenesis [64].

The greater risk of new onset T2DM in HF patients could be also the result of overactivity of the renin–angiotensin–aldosterone system (RAAS). In particular, increased angiotensin II (AT2) levels could be responsible for skeletal muscle vasoconstriction, which is associated with reduced glucose delivery and insulin sensitivity [65]. Moreover, AT2 is able to promote fibrosis, inflammation, apoptosis, and β-cell death in the pancreas, thus affecting insulin production [65]. The negative metabolic effects of RAAS could be also mediated by increased serum aldosterone levels [66–68]. Aldosterone, by activating mineralocorticoid receptors (MRs), can alter insulin sensitivity at the level of peripheral tissue [69] and can also induce inflammation at the level of pancreatic β‐cells, thus affecting insulin secretion [70].

Aside from sympathetic nervous system and RAAS overactivity, an altered glucose metabolism could also be related to the reduced activity of the natriuretic peptides (NPs) system [71]. NPs counteract RAAS and SNA [72] by promoting natriuresis and diuresis, exerting an antifibrotic effect at the cardiac level, causing vasodilation, and inhibiting RAAS. Although NP serum levels increase with the progression of HF, the effectiveness of natriuretic peptides is reduced because of an altered target organ responsiveness, overactivation of RAAS and SNA, and decreased availability of biologically active natriuretic peptides [73]. In obese patients or in those with metabolic syndrome, increased NP clearance could be related to an increased removal of the peptides by adipose tissue [74]. It is worth noting that in the presence of low serum levels of NPs, an increased risk of new onset T2DM has been observed [75]. This is due to the favorable metabolic effects of natriuretic peptides, NPs which are responsible for improved insulin sensitivity, increased lipolysis of brown fat, lipid oxidation, reduced ghrelin secretion, and reduction of the inflammatory status [73]. This last condition is also among the main factors that predispose a patient to the new onset of T2DM in HF due to the effects of cytokines and the related oxidative stress in HF [76].

Among the factors leading to the new onset T2DM in HF patients, limited physical activity and its impact on insulin sensitivity is worth noting. Reduced daily activity could be the result of non-adherence to an advised lifestyle, or it could be the consequence of the severity of HF. In both cases, reduced skeletal muscle perfusion and hypoxia lead to insulin resistance. Moreover, the loss of muscle mass could lead to a vicious cycle of worse functional limitations and insulin sensitivity [60–62].

Epidemiological data also demonstrate a greater incidence of new onset T2DM in patients affected by HF. In the Kaiser Permanente Northwest group, among 58,056 non-diabetic adults aged ≥ 30 years, HF was independently associated with an increase in T2DM at an incidence rate of 48% (95% confidence interval, CI, 27–73%); i.e., the incidence in HF diabetic patients is 13.6/1000 persons per year vs 9.2/1000 persons per year in non-diabetics [11]. In the analysis of a Danish registry involving 104,522 HF subjects with a first HF hospitalization and a mean follow-up of 3.9 years, 10% of the patients were diagnosed with new onset of T2DM. The incidence rate ranged from 2 per 100 persons per year in the first years up to 3 per 100 persons per year after 5 years of follow-up. Patients with new onset of T2DM also show a higher risk of death (HR 1.47; 95% CI 1.42–1.52) [77]. The greater incidence of T2DM in HF patients is also observed among elderly patients [78]. Moreover, the worse the functional capacity [79, 80] or the greater body mass index [81], the higher the risk of new onset T2DM.

## HF therapy, T2DM occurrence, and glycemic control

**Fig. 2:**
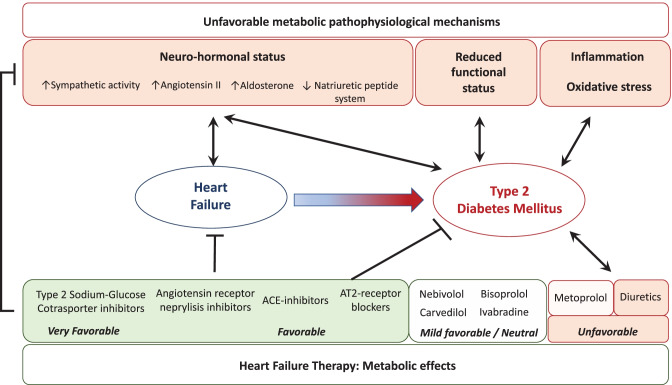
Pathophysiologic background leading to diabetes in heart failure patients and the influence of heart failure therapy on the risk of developing diabetes

Currently, different classes of drugs are recommended in order to improve the prognosis of HF patients, especially those with HFrEF [82]. The drugs are able to modulate neuro-hormonal system activity, control heart rate, and solve congestion. All these classes of drugs have been studied in order to evaluate their effects on glucose metabolism (Fig. 2).

**Beta-blockers** are the cornerstone of the therapeutic approach for HFrEF, because they counteract sympathetic nervous system overactivity and greatly improve patients’ prognosis. However, some studies have shown that the administration of beta-blockers could be associated with a worse glycemic status [83, 84]. The unfavorable metabolic effects are related to different mechanisms, such as the reduction of insulin secretion and insulin resistance [85]. Moreover, in the presence of a beta-blocker therapy, increased levels of catecholamines could activate alpha-adrenergic receptors and induce skeletal muscle vasoconstriction, leading to skeletal muscle hypoperfusion and reduction in glucose uptake [85]. This condition could be even more evident in therapies using a non-selective beta-blocker, due to the loss of beta-2 adrenergic receptor-mediated vasodilation [85]. However, beta-blockers are a heterogeneous class of drugs and the most recent ones, due to their ancillary actions, do not have the same unfavorable metabolic effects [86–89]. Carvedilol is a non-selective blocker of beta-adrenergic receptors and is also able to block alpha-adrenergic receptors, thus increasing skeletal muscle perfusion [88]. Moreover, carvedilol is able to reduce oxidative stress [88]. These mechanisms could explain the ability of carvedilol to exert a more favorable metabolic effect than metoprolol in the COMET trial [86]. In 2,298 patients without T2DM at baseline, a new onset T2DM was observed more frequently in the metoprolol group than in the carvedilol group (12.6 vs 10.3; HR: 0.78; 95%CI: 0.61–0.997; *p*: 0.048) [86]. Moreover, a greater incidence of diabetic events (diabetic coma, peripheral gangrene, diabetic foot, decreased glucose tolerance, or hyperglycemia) occurred in the metoprolol group (13 vs 10.6%; HR: 0.78; 95%CI: 0.61–0.99; *p*: 0.039). These results are further supported by the GEMINI trial, in which carvedilol and metoprolol effects in hypertensive diabetic patients were evaluated [88]. HbA1c increases with metoprolol but not with carvedilol, whereas insulin sensitivity improves with carvedilol but not metoprolol. Finally, the progression of microalbuminuria is less frequent with carvedilol. Nebivolol could have a more favorable metabolic profile [89]. Nebivolol is a selective beta-1 adrenergic receptor blocker which has, among other ancillary effects, nitric oxide-mediated vasodilatory and antioxidative properties [89]. Finally, bisoprolol has demonstrated to have a neutral effect on the glycemic control [90].

### ACE inhibitors, angiotensin receptor blockers, and sacubitril/valsartan

Both angiotensin-converting enzyme inhibitors (ACEi) and angiotensin receptor blockers (ARBs) have been demonstrated to not only improve the prognosis in patients with HFrEF [91] but also to reduce the number of new onset T2DM cases [82–93]. These results are in line with previous studies of hypertensive patients that have demonstrated the ability of RAAS inhibitors to prevent T2DM [94, 95]. The mechanisms by which RAASi exert their protective effects against T2DM are not completely known. RAASi are responsible for vasodilatory effects, mediated by prostaglandin and nitric oxide pathways that lead to an improvement in skeletal muscle blood flow and glucose uptake [96]. ACEi could potentiate these effects by inhibiting the degradation of bradykinin [97]. Finally, the inhibition of AT2 vasoconstrictive effect in the pancreas increases islet blood flow, thus improving insulin release [98].

The favorable metabolic effects of valsartan are enhanced by the concomitant inhibition of neprilysin by sacubitril. Sacubitril/valsartan is able to further improve HFrEF prognosis when compared with enalapril [99], and it has been associated with better control of glycemic status [100] compared to enalapril. This effect could be caused by the inhibition of neprilysin and the increased availability of natriuretic peptides, which, as mentioned previously, improve metabolic status [71]. Moreover, it has been hypothesized that sacubitril, by inhibiting neprilysin, is also able to reduce the degradation of type 1 glucagon-like peptide (GLP1), thus further improving glycemic control [101].

### Mineralcorticoid antagnists (MRA)

have demonstrated to improve outcome and are currently recommended to treat HFrEF patients [82]. A retrospective analysis from EPHESUS revealed that eplerenone reduces the risk of CV mortality by 17% in diabetic patients and the absolute risk reduction was much more significant in diabetic vs non diabetic subjects [102].

Although some concerns have been hypothesized about potential relation between MRA and increased incidence of T2DM, the EMPHASIS trial showed the eplerenone administration has neutral effects on new onset diabetes in HFrEF [103]. Moreover, in a TOCAT sub-analysis, in T2DM patients with LVEF ≥ 45%, the extracellular matrix deposition is overexpressed and the spironolactone treatment attenuates inflammatory and profibrotic biomarkers [104]. Overall, these findings demonstrate an optimal safety and additional positive effects of MRA in patients with both T2DM and HF.

### Diuretics

In contrast to sacubitril/valsartan, thiazides and loop diuretic effects are unfavorable from a metabolic point of view [105–107] because they lead to impaired glucose tolerance, which is probably related to insulin resistance. This is a dose-dependent relationship that is reversible after diuretic withdrawal. The hyperglycemic effects could be related to hypokalemic effects [108] or to increased hepatic fat [109].

The association of loop diuretic dosage (furosemide equivalents) 90 days after discharge with risk of T2DM was evaluated in a Danish registry of 99,362 patients [110]. The study found that loop diuretic dosages are associated with an increased risk of developing T2DM in a dose-dependent manner. Concomitant use of renin-angiotensin system inhibitors attenuates the risk [110].

### Ivabradine

In this case "Ivabradine" as indicated of persistence of a high sinus heart rate after uptitration of beta-blockers, the use of ivabradine is recommended in order to control heart rate [82]. In a group of patients with coronary artery disease, the administration of ivabradine was found to be associated with a reduction of fasting glucose levels and glycated hemoglobin [111]. These data support the possible neutral or favorable effect of ivabradine on glucose metabolism.

## Conclusion

The data from experimental, observational, and randomized control trials clearly demonstrate the double link between T2DM and HF. Patients with T2DM present an increased risk of new onset of HF as well as HF-related events. On the other hand, HF patients present an increased risk of new onset T2DM. Both HF in T2DM and T2DM in HF are associated with a worse prognosis. These data are even more relevant considering the new therapeutic opportunities derived by the administration of SGLT2i [15, 16, 112–114].
